# CRISPR/Cas9-targeted *smpB* mutation revealing roles in biofilm formation, motility, and antibiotic susceptibility in *Acinetobacter baumannii*

**DOI:** 10.1371/journal.pone.0329638

**Published:** 2025-08-04

**Authors:** Techit Thavorasak, Sirijan Santajit, Witawat Tunyong, Thida Kong-Ngoen, Onrapak Reamtong, Sumate Ampawong, Nawannaporn Saelim, Thapani Srisai, Pisinee Aiumurai, Pornpan Pumirat, Wanpen Chaicumpa, Nitaya Indrawattana

**Affiliations:** 1 Department of Microbiology and Immunology, Faculty of Tropical Medicine, Mahidol University, Bangkok, Thailand; 2 Department of Medical Technology, School of Allied Health Sciences, Walailak University, Tha Sala, Thailand; 3 Department of Tropical Molecular Biology and Genetics, Faculty of Tropical Medicine, Mahidol University, Bangkok, Thailand; 4 Department of Tropical Pathology, Faculty of Tropical Medicine, Mahidol University, Bangkok, Thailand; 5 Siriraj Center of Research Excellence in Allergy and Immunology, Department of Research, Faculty of Medicine Siriraj Hospital, Mahidol University, Bangkok, Thailand; 6 Center of Excellence in Therapeutic Proteins and Antibody Engineering, Department of Parasitology, Faculty of Medicine Siriraj Hospital, Mahidol University, Bangkok, Thailand; 7 Biodesign Innovation Programm, Department of Parasitology, Faculty of Medicine Siriraj Hospital, Mahidol University, Bangkok, Thailand; 8 Biomedical Research Incubator Division, Department of Research, Faculty of Medicine Siriraj Hospital, Mahidol University, Bangkok, Thailand; AIIMS: All India Institute of Medical Sciences, INDIA

## Abstract

**Background:**

*Acinetobacter baumannii* is a multidrug-resistant pathogen and a major cause of hospital-acquired infections worldwide. Its ability to survive in harsh environments and evade antibiotic treatments underscores the urgent need for new therapeutic targets. Emerging evidence suggests that the small protein B (SmpB) may also play broader roles in bacterial virulence, including regulation of biofilm formation, motility, and stress adaptation. However, the specific contributions of SmpB to these pathogenic traits in *A. baumannii* remain poorly defined. Addressing this knowledge gap is essential for evaluating SmpB as a potential antimicrobial target and developing new strategies to combat multidrug-resistant infections.

**Methods:**

CRISPR/Cas9-mediated gene editing was used to generate a targeted *smpB* mutant in *A. baumannii*. The *smpB* mutant was assessed for growth, biofilm formation, motility, antibiotic susceptibility, and virulence. Biofilm was quantified via crystal violet staining and microscopy, while motility was examined using swimming, swarming, and twitching assays. Antibiotic susceptibility was evaluated using disk diffusion. Virulence was tested in the *Galleria mellonella* infection model. Proteomic analysis was performed to identify changes in protein expression associated with *smpB* disruption,

**Results:**

CRISPR/Cas9-mediated editing successfully introduced a C212T nucleotide substitution in the smpB gene, resulting in an A89G amino acid change. Growth curve analysis showed no significant difference between the wild-type and *smpB* mutant strains under nutrient-rich conditions. However, the mutant exhibited a significant reduction in biofilm formation (*p* = 0.0079) and impaired twitching motility, while swimming and swarming motility remained unaffected. Antibiotic susceptibility testing revealed increased sensitivity to ceftizoxime, piperacillin/tazobactam, and gentamicin, alongside decreased susceptibility to cefepime, tetracycline, and spectinomycin. In the *G. mellonella* infection model, the *smpB* mutant showed reduced virulence, with 84% larval survival compared to 72% in the wild type (*p* = 0.4183). Proteomic analysis revealed downregulation of key stress response and virulence-associated proteins, including GroEL, DnaK, RecA, and PirA, while proteins involved in ribosome maturation and transcription, such as RimP and RpoA, were upregulated. STRING network analysis supported the broad regulatory role of SmpB in biofilm formation, motility, stress adaptation, and pathogenesis.

**Conclusion:**

This study demonstrates that SmpB is a key regulator of biofilm formation, twitching motility, antibiotic response, and virulence in *A. baumannii*. While not essential for growth under optimal conditions, *smpB* disruption impairs multiple pathogenic traits and alters stress-related proteomic pathways. These findings highlight the potential of SmpB as a novel antimicrobial target, offering a promising strategy to weaken bacterial virulence without promoting resistance. Targeting the trans-translation system may pave the way for innovative therapies against multidrug-resistant *A. baumannii*.

## 1. Introduction

*Acinetobacter baumannii* is an opportunistic pathogen behind severe hospital-acquired infections. It possesses an open and highly plastic pan-genome, comprising both core and accessory genes that contribute to its adaptability and multidrug resistance. The pan-genome harbors a diverse array of virulence factors, antibiotic resistance determinants, and regulatory elements that facilitate survival in hostile environments known for its multidrug resistance and adaptability to harsh environments [1]. A key factor in its survival is the ability to resolve ribosome stalling, which disrupts protein synthesis when ribosomes encounter damaged or truncated mRNAs. To overcome this, *A. baumannii* relies primarily on trans-translation, a rescue mechanism involving transfer-messenger RNA (tmRNA) and the small protein B (SmpB). SmpB binds tmRNA, facilitating its interaction with stalled ribosomes, enabling translation to resume on the tmRNA template and marking the incomplete peptide for degradation [2–4]. The disruption of *smpB* leading to an accumulation of stalled ribosomes and incomplete proteins, which can be detrimental to the cell [5–7]. Studies have shown that editing *smpB* gene results in severe growth defects and increased sensitivity to stress conditions in bacteria [8–10]. The ribosome rescue mechanisms, including both trans-translation and alternative pathways, are absent in eukaryotes, making them attractive targets for novel antibacterial therapies. By targeting these bacterial-specific processes, it is possible to develop new antibiotics that selectively inhibit bacterial growth without affecting human cells. This approach holds promise for addressing the growing issue of multidrug-resistant *Acinetobacter* infections [11].

Currently, CRISPR/Cas9 techniques are being applied in the medical sciences, the genetic alterations of bacteria, fungi, laboratory animals and plants [12–13] make the CRISPR-Cas9 technique an interesting one in the study. Components of the CRISPR/Cas9 are short guide RNA (sgRNA), Cas9 nuclease, target gene, and specific base sequence (called protospacer-adjacent motif or PAM) of the target DNA location. A Cas9-induced double strand break (DSB) in the genome is lethal to most bacteria because they do not possess a non-homologous end-joining (NHEJ) repair pathway, which is the major pathway for DSB repair in eukaryotic cells [14]. Only cells whose genomes are repaired by the homologous recombination (HR) pathway can survive [15]. Therefore, it is possible to achieve one-step scarless genome editing in *A. baumannii* by taking advantage of the CRISPR-Cas9 genome cleavage system.

This study aims to characterize growth, motility, biofilm forming capacity, antibiotic resistance, virulence, and proteomic profiles of the *A. baumannii* that *smpB* gene have been interrupted by the CRISPR-Cas9 technique. The information paves a way for a rational design of the new anti-bacterial class (novel targets, i.e., ribosome rescue apparatus) against the drug resistant *A. baumannii* including ESKAPE and other pathogenic bacteria.

## 2. Materials and methods

### 2.1 Bacteria strains and growth conditions

*Acinetobacter baumannii* ATCC17978 was purchased from American Type Culture Collection. *Escherichia coli* strain DH5α was obtained from Professor Dr. Wanpen Chaicumpa (Center of Research Excellence in Therapeutic Proteins and Antibody Engineering, Department of Parasitology, Faculty of Medicine Siriraj Hospital, Mahidol University, Bangkok, Thailand). pBECAb-apr was a gift from Quanjiang Ji (Addgene plasmid # 122001; http://n2t.net/addgene:122001; RRID:Addgene_122001).

### 2.2 Gene editing procedure

To interrupt the *smpB* gene of *A*. *baumannii*, gene-specific sgRNAs for *smpB* system gene of the bacteria were designed using CHOPCHOP web tool [16]. The sgRNA that contains a targeting sequence [crRNA sequence; Spacer-Fˊ(5ˊ-tagtTTTCGTGTACGTGTAGCTTC-3ˊ) and Rˊ(5ˊ-aaacGAAGCTACACGTACACGAAA-3ˊ)] were synthesize commercially (Integrated DNA Technologies, IA, USA). Briefly, the synthetic oligonucleotides were subjected for phosphorylation with T4 Polynucleotide Kinase (New England Biolabs, MA, USA). The phosphorylated products were annealed and then cloned into pBECAb-apr plasmid (Addgene, MA, USA) in the present of T4 DNA Ligase buffer, *BsaI*-HFv2, and T4 DNA ligase (New England Biolabs). The Golden Gate ligation was then performed inside a thermocycler using the parameter: 25 cycles at 37°C for 3 min, and 16°C for 4 min; 50°C for 5 min; 80°C for 10 min; hold at 16°C. Ten microlitre of ligation product were transformed into 100 µL of DH5α *E. coli* competent cells using heat shock transformation. The transformed cells were plated onto an LB agar plate supplemented with 50 μg/mL of apramycin (LB-apr agar) and the plate was incubated at 37°C for 16 hours. The transformants were picked randomly and subjected to direct colony PCR with the Spacer-Fˊ and M13R (5ˊ-CAGGAAACAGCTATGACC-3ˊ) primers to verify the successful cloning of the spacer sequence (sgRNA) into pBECAb-apr plasmid. The PCR reaction mixture contained 1.25 µL of 10 × *Taq* DNA polymerase buffer (Thermo Fisher Scientific, MA, USA), 0.75 µL of 25 mM MgCl_2_, 0.5 µL of 10 mM dNTPs, 0.25 µL of 10 µM Spacer-Fˊ, 0.25 µL of 10 µM M13R, 0.1 µL of *Taq* DNA polymerase, and 9.4 µL of molecular water. The amplification was performed, and the amplicons were subjected to an agarose gel electrophoresis to determine the size of amplicons, of which the theoretical size is 224 bp. The positive clones were inoculated into 5 mL of LB broth containing 50 μg/mL Apramycin and incubated at 37°C for 16 hours with shaking at 250 rpm. The spacer-introduced pBECAb-apr plasmids were extracted by using FavorPerpTM plasmid extraction mini kit (Favorgen Biotech Corporation, Ping-Tung, Taiwan) according to the manufacturer protocol and determined the concentration by using NanoDrop (Thermo Fisher Scientific). The spacer sequence introduced in pBECAb plasmid was verified by DNA sequencing using M13R primer. The verified spacer-introduced pBECAb-apr plasmids were transformed into *A. baumannii* by electroporation. The transformed cells were plated onto an LB-apr agar plate and incubated at 37°C for 16 hours. The transformants were picked randomly and subjected to direct colony PCR with the Spacer-Fˊ and M13R primers as mentioned above. Positive transformants colonies, bacterial colony that carries spacer-introduced pBECAb-apr plasmids, were inoculated into 5 mL of LB broth and incubated overnight at 37°C with shaking for 16 hours for plasmid curing. Afterward, the bacterial culture was streaked onto LB agar containing 5% sucrose and incubated at 37°C for 16 hours. Then, bacterial colonies were randomly picked and streaked onto LB and LB-apr agars, respectively to identify the clone that completes spacer-introduced pBECAb-apr plasmid curing, *smpB* mutant strain [17]. Then, the selected colony was inoculated into 3 mL of LB broth and incubated overnight at 37°C with shaking for 16 hours. The bacterial culture was diluted in molecular water (1:10), boiled for 10 min and used as the template for *smpB* amplification. The PCR was performed using Q5® High-Fidelity DNA Polymerase (New England Biolabs) with *smpB* specific primers. The amplicons were then subjected to agarose gel electrophoresis. The PCR product band was excised from agarose gel and purified using GenepHlow™ Gel/PCR Kit (Geneaid, New Taipei City, Taiwan). The purified PCR product was then subjected to DNA sequencing with *smpB* specific primers to determine the mutation of *smpB* sequences. The sequences were aligned using Jalview software version 2.11.3.3 [18].

### 2.3 Growth curve analysis

Growth curves of *A. baumannii* WT and *smpB* mutant strains were determined as previously described [19] by growing them overnight at 37°C in LB broth with shaking aeration 200 rpm. The overnight cultures were adjusted to one optical density (OD) at a wavelength of 600 nm in LB broth. One mL of the OD-adjusted bacterial was inoculated into 50 mL of LB broth and incubated at 37°C with shaking (160 rpm) for 9 hours. OD at 600 nm was determined every hour by 100 µL sample of every culture. Data obtained from three independent cultures grown on the same day of individual bacterial strains was averaged and presented as the mean ± SD.

### 2.4 Biofilm formation capacity and cell morphology

Overnight cultures of *A. baumannii* WT and *smpB* mutant strains were diluted 1:100 in fresh LB broth and grown to mid-log phase (OD_600nm_ ~ 0.5). The cultures were then further diluted to an OD_600nm_ of 0.05 in LB broth. Biofilm formation ability was assessed using two techniques: crystal violet staining and scanning electron microscopy (SEM) according to the previous study [19]. Cell morphology was examined using transmission electron microscopy (TEM).

For the crystal violet staining method, 200 µL of diluted bacterial cultures were added to the wells of a sterile, flat-bottom 96-well polystyrene microtiter plate. The plates were incubated at 37°C for 24 hours without shaking to allow biofilm formation. After incubation, the wells were gently washed three times with 200 µL of phosphate-buffered saline (PBS) to remove non-adherent cells. The wells were then stained with 200 µL of 0.1% crystal violet solution for 15 minutes at room temperature. Excess stain was removed by washing the wells three times with 200 µL of distilled water. The bound crystal violet was solubilized with 200 µL of 95% ethanol and the absorbance of the solubilized dye was measured at 570 nm using a microplate reader. All experiments were performed in triplicate and repeated at least three times. Wells containing sterile LB broth without bacteria were used as negative controls to account for background crystal violet staining. The WT strain served as the positive control for biofilm formation.

For SEM method, briefly, 200 µL of bacterial culture was inoculated into a 24-well plate containing an SEM blocking film and incubated at 37°C overnight. After incubation, wells were washed with PBS, followed by fixation with 2.5% glutaraldehyde in 0.1 M sucrose phosphate buffer (SPB) at pH 7.4 overnight at 4°C. The samples were then washed with SPB, post-fixed with 1% osmium tetroxide (OsO_4_) in 0.1 M SPB, dehydrated through a graded ethanol series, and dried using a critical point drying machine (Leica EM CPD300, Germany). The dried samples were coated with a 10 nm thick gold-palladium film (Quorum Q150R-S Plus, UK) and examined using a scanning electron microscope (JSM-6610LV; JEOL Ltd., Tokyo, Japan).

For the TEM assay, cultures were grown overnight in LB broth at 37°C, centrifuged, and resuspended in 2.5% glutaraldehyde in 0.1 M SPB at 4°C overnight. Cells were then washed, post-fixed with 1% OsO_4_ in 0.1 M SPB, and stained with 2% uranyl acetate before examination with a TEM (Hitachi HT7700, Japan). These procedures provided detailed visualization of biofilm formation and cell morphology, offering insights into the structural characteristics of *A. baumannii*.

### 2.5 Surface motility

*A. baumannii* was known for their ability to surface-associated motility, which plays a critical role in biofilm formation and colonization of new environment. The ability to surface-associated motility of *A. baumannii* WT and *smpB* mutant strains was determined following the established protocol [19]. For swimming and swarming motility, overnight cultures grown in Luria-Bertani (LB) broth at 37°C with shaking at 200 rpm were used to inoculate LB plates containing 0.3–0.5% (w/v) agar. A 10 µL aliquot of culture was spotted at the center of the agar surface, and plates were incubated at 30°C or 37°C for 24 hours. The diameter of the motility halo was measured to assess bacterial movement across the surface. For twitching motility, LB agar plates containing 1.0% agar were prepared. A sterile toothpick was used to stab each bacterial culture through the agar to the bottom of the Petri dish, ensuring contact with the plastic surface. Plates were incubated upright at 30°C for 24–48 hours. After incubation, the twitching zone was measured to evaluate the extent of type IV pilus-mediated surface motility. Non-inoculated plates were used as negative controls to confirm the absence of spontaneous motility, and the WT strain was used as the positive control for all motility assays.

### 2.6 Antibiotic susceptibility testing

The antimicrobial sensitivity of *A. baumannii* was determined using the minimum inhibitory concentration (MIC) method based on the Epsilometer test (E-test). This method involved testing a range of antimicrobial agents, including amplicilin, ceftizoxime, cefepime, cefoperazone/sulbactam, gentamicin, imipenem, kanamycin, meropenem, penicillin G, piperacillin/tazobactam, spectimomycin, streptomycin, and tetracycline (Liofilchem, Roseto degli Abruzzi, Italy). To conduct the tests, bacterial cultures were prepared by inoculating a single colony of *A. baumannii* into Mueller-Hinton broth and incubating overnight at 37°C with shaking. The cultures were then adjusted to a turbidity equivalent to 0.5 McFarland standard and spread evenly onto Mueller-Hinton agar plates. E-test strips impregnated with a gradient of the antimicrobial agents were placed on the inoculated agar surfaces and incubated at 37°C for 24 hours. MIC values were read at the point where the elliptical zone of inhibition intersected the E-test strip. The results were interpreted according to the guidelines of the Institute for Clinical and Laboratory Standards (CLSI) version 2020 [20]. Each assay was performed in triplicate to ensure accuracy and reproducibility. The WT strain was used as a positive control for comparison of MIC values, and sterile Mueller-Hinton agar served as the negative control to ensure absence of contamination or background inhibition.

### 2.7 *Galleria mellonella* infection assay

The *G. mellonella* caterpillar model was employed to investigate the toxicity and virulence of *A. baumannii* as mentioned in the previous study [19]. The protocol for *A. baumannii* infection in wax moth larvae was approved by the Animal Ethics Committee, Faculty of Tropical Medicine, Mahidol University, Bangkok, Thailand (Reference No: FTM—ACUC 011/2023). Forty larvae, each measuring 2–2.5 cm in length and weighing 200–250 mg, were selected for the experiment, ensuring they were free from melanization. After 18 hours of bacterial growth, the bacterial culture was diluted in PBS to a concentration of approximately 3 × 10^8^ CFU/mL by adjusting the OD_600nm_. A Hamilton syringe was used to inject 10^5^ CFU of the bacterial suspension into the body cavity of each larva via the proleg. Control larvae received PBS injections. Post-injection, the larvae were incubated in the dark at 37°C for 5 days and monitored daily for viability. The day after injection, individual larvae were assessed for pigmentation changes and mobility. Larvae were deemed dead if they did not respond to gentle prodding with a pipette tip. The number and time of death for each larva were recorded, and a survival graph was subsequently plotted.

### 2.8 Protein extraction and preparation

Bacteria were disrupted using a sonicator in a lysis buffer containing 8 M urea. Protein concentration was determined using a BCA protein assay. Subsequently, proteins were treated with 10 mM DTT at 37°C for 1 h for reduction and then with 40 mM iodoacetamide (IAA) at room temperature in the dark for 1 h for alkylation. Excess IAA was quenched with 40 mM DTT at room temperature in the dark for 15 min. The lysate was then digested with trypsin overnight at 37°C. Finally, the resulting peptides were desalted using a C18 reverse-phase column and dried prior to LC-MS/MS analysis. Proteomics analysis was performed in triplicate to ensure the consistency and reliability of results. Each replicate involved independent preparation of bacterial cultures, protein extraction, and digestion under identical conditions. Mass spectrometry was conducted separately for each replicate, and data were analyzed individually before integration. Proteins with consistent expression patterns across all three replicates were considered robust and reliable for downstream analysis.

### 2.9 LC-MS/MS analysis

For LC-MS/MS protein identification, a Q Exactive Plus mass spectrometer was utilized. Bacteria from two experimental groups were compared quantitatively using stable isotopic dimethyl labeling (light labeling for the WT group and heavy labeling for the mutant group). After labelling, samples were mixed and analyzed by LC-MS/MS. The method involved a full MS scan at a resolution of 70,000 followed by 10 data-dependent MS2 scans at 17,500 resolution. The collision energy for high-energy collision dissociation (HCD) fragmentation was set at 32%. Specific parameters excluded precursor ions with unassigned charge states, a charge state of +1, or a charge state greater than +8. A dynamic exclusion of 30 seconds was applied.

MS raw data files were processed using Proteome Discoverer™ Software 2.1, searching against a composite database containing forward and reversed peptide sequences of the *A. baumannii* Swiss-Prot Database. Search parameters included fixed modifications (carbamidomethylation of cysteine and dimethylation of N-termini and lysine residues for light and heavy labelling) and a variable modification (oxidation of methionine). Parent and fragment mass errors were limited to 10 ppm. A target-decoy approach was used to maintain a false discovery rate of identified peptides below 5%. Protein-protein interaction was analyzed by STRING database.

### 2.10 Statistical analysis

Statistical analyses were performed to ensure the reproducibility and significance of experimental results. All experiments were conducted in at least three independent biological replicates, and data are presented as the mean ± standard deviation (SD), unless otherwise specified. For comparisons between two groups (e.g., WT vs *smpB* mutant), an unpaired two-tailed Student’s t-test was used. For comparisons among more than two groups, one-way analysis of variance (ANOVA) followed by Tukey’s post hoc test was applied. MIC values, motility zone diameters, and biofilm quantification were statistically compared between groups using appropriate tests as described in the Results section. For survival analysis in the *Galleria mellonella* infection model, Kaplan–Meier survival curves were generated, and statistical differences were assessed using the log-rank (Mantel–Cox) test. A *p*-value < 0.05 was considered statistically significant in all analyses. All statistical tests and data visualizations were performed using GraphPad Prism version 9 (GraphPad Software, San Diego, CA, USA), a validated and widely accepted tool for biomedical statistical analysis.

## 3. Results

### 3.1 Gene mutation confirmation and sequencing analysis

The designed sgRNAs were introduced into *A. baumannii* ATCC19606 using a CRISPR-Cas9 system. The efficiency of gene editing was confirmed by PCR and sequencing of the targeted gene region (S1 Fig). The sequencing results confirmed successful mutations in the tmRNA/smpB gene. The results revealed a nucleotide substitution from C to T at position 212 (C212T), resulting in an amino acid change from alanine to glycine at position 89 (A89G) (S1 and S2 Figs). The result showed a single base change in the target sequence and the presence of the anticipated mutations at the site.

### 3.2 Growth analysis of *Acinetobacter baumannii* WT and *smpB* mutant strains

**Fig 1:**
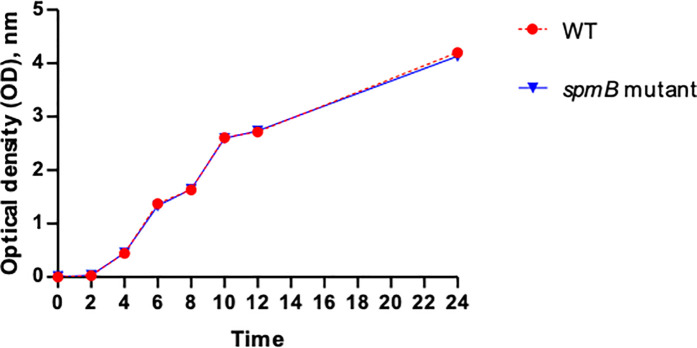
Growth characteristics of *A. baumannii* strains used in this study. Wild type (*A. baumannii* ATCC17978) and *smpB* mutant strains were cultured in LB broth at 37°C with shaking. Optical density (OD) was measured at 600 nm. Data points and error bars represent the mean ± SEM from three independent experiments.

The growth characteristics over time in *A. baumannii* WT compared to *smpB* mutant were investigated, and the results are illustrated in Fig 1. The analysis revealed there was no significant difference in the doubling times between the WT and *smpB* mutant strains (*p* > 0.05), indicating that the *smpB* mutation does not substantially affect the overall growth pattern.

### 3.3 Biofilm production and cell morphology in WT and *smpB* mutant strains

**Fig 2:**
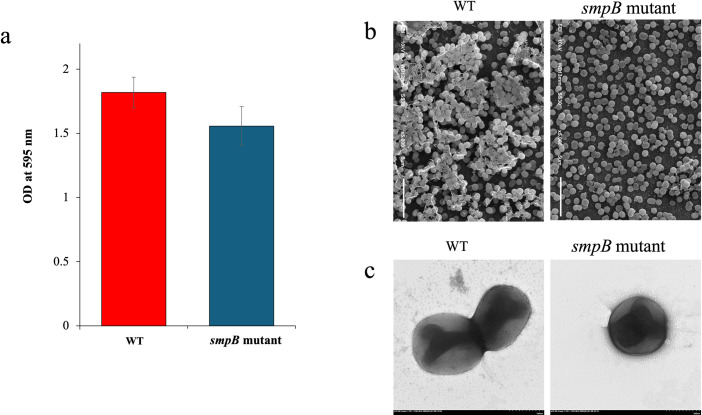
Comparative analysis of biofilm formation and morphology in *A. baumannii.* (a) Biofilm formation quantification using crystal violet staining, comparing WT and *smpB* mutant strains. (b) Scanning electron microscopy (SEM) images showing biofilm structure and surface coverage. The left panel represents the WT strain, while the right panel shows the *smpB* mutant strain. (c) Transmission electron microscopy (TEM) images of negatively stained cells. The left panel represents the WT strain, and the right panel shows the *smpB* mutant strain. WT refers to the wild-type strain, and *smpB* mutant indicates the strain with a mutation in the *smpB* gene.

Biofilm production of both WT and *smpB* mutant bacterial groups was quantified by measuring the optical density at 595 nm following biofilm crystal violet staining, as shown in Fig 2a. The WT group exhibited a mean OD_595nm_ of 1.818 ± 0.120, while the mutant showed a lower mean OD_595nm_ of 1.556 ± 0.152. A two-sample t-test revealed a statistically significant difference between the two groups (*p* = 0.0079), indicating that the WT group has a significantly higher biofilm production compared to the mutant strain. This significant difference suggests that the *smpB* mutation is associated with a reduced biofilm production capability.

The cell morphology of *A. baumannii* WT and *smpB* mutant strains was analyzed using SEM and TEM. SEM analysis revealed notable differences in the biofilm architecture between the WT and *smpB* mutant strains (Fig 2b). The WT strain exhibited dense, multilayered biofilms, suggesting a robust ability to adhere and form structured communities, whereas the *smpB* mutant strain formed sparser, less organized biofilm structures, indicating a reduced capacity for biofilm production. TEM analysis further highlighted these differences at the cellular level. The WT strain displayed intact, well-defined cell envelopes, supporting the structural integrity necessary for biofilm development. While the *smpB* mutant also maintained intact cell envelopes, the disorganization of its biofilm suggests that the *smpB* gene plays a critical role in regulating biofilm formation, possibly by influencing factors that enhance cell-to-cell interactions and adhesion.

### 3.4 Altered antibiotic susceptibility profiles in *smpB* mutant strains

The minimum inhibitory concentration (MIC) values of various antibiotics were determined for *A. baumannii* WT and its *smpB* mutant to assess the impact of the *smpB* gene mutation on antibiotic susceptibility. MIC values for ampicillin, cefoperazone/sulbactam, imipenem, meropenem, kanamycin, and streptomycin were not significantly different between WT and *smpB* mutant strains (*p* > 0.05, unpaired t-test). However, the *smpB* mutant exhibited significantly increased susceptibility to ceftizoxime (**p* *= 0.020), piperacillin/tazobactam (*p* = 0.041), and gentamicin (*p* = 0.013), as determined by unpaired *t*-*t*est. In contrast, the mutant showed decreased susceptibility to cefepime (*p* = 0.038), tetracycline (*p* = 0.047), and spectinomycin (*p* = 0.012). These findings indicate that disruption of *smpB* impacts the response to selected antibiotics (Table 1).

**Table 1:** Minimal inhibitory concentration of antibiotics used in this study.

Antibiotic	MIC (μg/ml)	
	*A. baumannii* ATCC17978 (WT)	*A. baumannii smpB* mutant
Penicillin G (P)	>32	>32
Amplicilin (A)	24	24
Ceftizoxime (CZX)	8	6
Cefepime (FEP)	2	3
Piperacillin/tazobactam (TZP)	1	0.75
Cefoperazone/sulbactam (CPS)	0.75	0.75
Imipenem (IMI)	0.25	0.25
Meropenem (MRP)	0.25	0.25
Gentamicin (CN)	0.75	0.5
Kanamycin (K)	1	1
Spectimomycin (SPC)	32	64
Streptomycin (S)	12	12
Tetracycline (TE)	3	4

### 3.5 Surface motility

**Fig 3:**
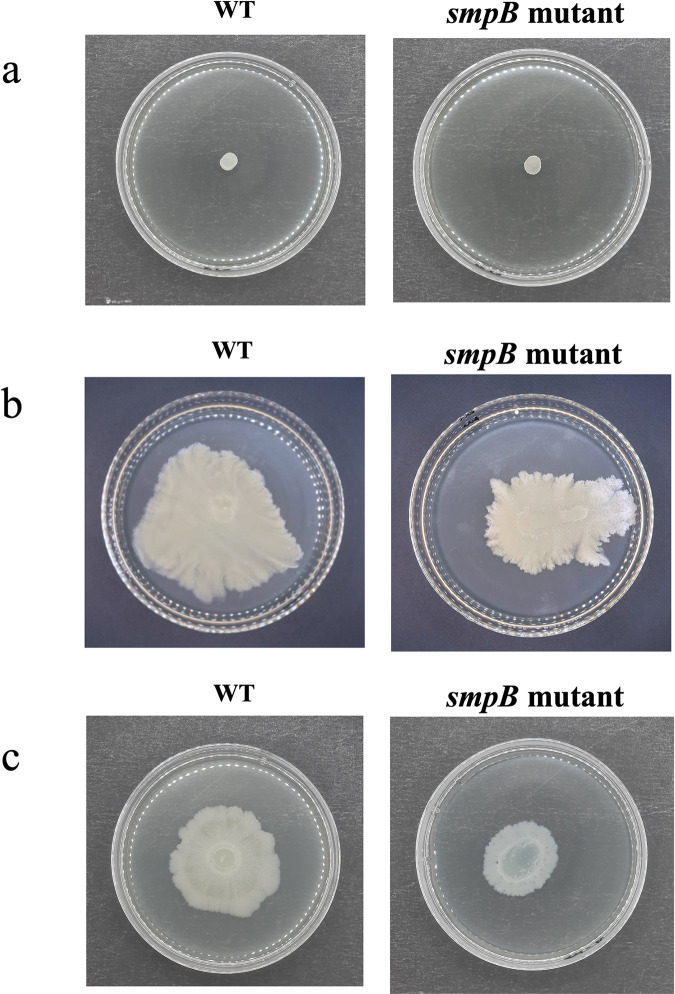
Surface motility assays illustrating the swimming. (a), swarming (b), and twitching (c) capabilities of *A. baumannii*. (a) Swimming motility is demonstrated by the diameter of the circular zone of bacterial migration from the inoculation point on a semisolid agar plate. (b) Swarming motility is observed through the expansion of bacterial growth across the surface of a nutrient-rich agar medium. (c) Twitching motility is shown by the formation of spreading zones beneath the agar surface due to bacterial movement. WT represents the wild-type strain, while *smpB* mutant refers to the strain with the smpB gene mutation.

The ability of *A. baumannii* to move across surfaces is critical for biofilm formation and colonization of new environments. The surface motility of *A. baumannii* WT and *smpB* mutant strains was assessed by measuring the diameter of the motility halo formed around the inoculation spot on motility agar plates. The results revealed no significant differences in swimming (mean halo diameter: WT 21.2 ± 1.4 mm vs *smpB* mutant 20.8 ± 1.7 mm; *p* = 0.615) and swarming motility (WT 18.5 ± 1.2 mm vs *smpB* mutant 17.9 ± 1.6 mm; *p* = 0.477), as analyzed using unpaired *t*-tests (Figs 3a and 3b). In contrast, the *smpB* mutant exhibited significantly reduced twitching motility (WT 11.4 ± 0.9 mm vs *smpB* mutant 7.2 ± 1.1 mm; *p* = 0.009), indicating that SmpB plays a critical role in regulating type IV pilus-mediated surface movement (Fig 3c).

### 3.6 Pathogenicity of *smpB* mutant in *Galleria* infection model

**Fig 4:**
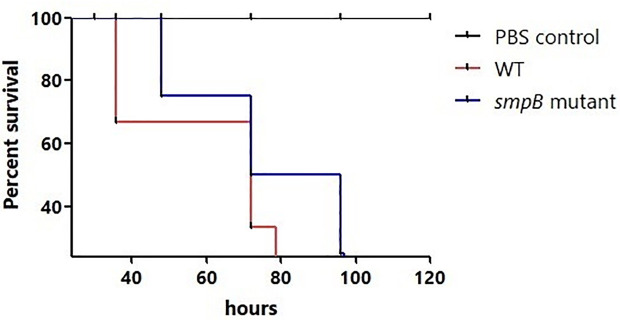
Percentage survival of *Acinetobacter baumannii* infected *Galleria mellonella.* Survival of *G. mellonella* infected with *A. baumannii* wild type, *smpB* mutant, or PBS control. Data represent means of three independent experiments, with error bars showing standard deviations. WT: wild-type strain; *smpB* mutant: strain with smpB gene mutation.

In the investigation of pathogenicity differences between the *A. baumannii* WT strain and its *smpB* mutant counterpart utilizing the *G. mellonella* infection assay, the Kaplan-Meier plot illustrates the mean survival rates of the infected larvae (Fig 4). The larvae infected with the *A. baumannii* WT strain show a steady decrease in survival over time after 36 hours of infection, indicating pathogenicity of bacteria. In contrast, the larvae infected with the *smpB* mutant strain exhibit a slower decrease in survival, indicating reduced pathogenicity. Statistical analysis revealed a difference in survival rates between *A. baumannii* WT and *smpB* mutant strains, 72% survival and 84% survival, respectively (*p* = 0.4183). Although there is no significant difference in the percent of survival rate, but the decreased survival rate of mutant suggested that smpB gene in *A. baumannii* possibly reduces its pathogenicity in *G. mellonella*, as inferred from the survival rates observed in this study. Further research may explore the role of *smpB* in other aspects of *A. baumannii* pathogenicity and survival.

### 3.7 Comprehensive proteomic analysis of *smpB* mutant in *Acinetobacter baumannii* compared to WT strain

Quantitative proteomics revealed significant alterations in protein expression, particularly in pathways associated with biofilm formation, motility, and virulence (S1). Key downregulated proteins in the *smpB* mutant included chaperones GroEL and DnaK, essential for protein folding and stress response, and critical for biofilm stability [21]. This downregulation correlates with reduced biofilm production, as evidenced by crystal violet staining and microscopy analysis, which revealed sparse and less cohesive biofilm architecture in the *smpB* mutant. Proteins related to motility, such as ATP synthase subunit b (AtpF) and elongation factor G (FusA), were also downregulated, consistent with impaired twitching motility observed in the mutant. Furthermore, the downregulation of RecombinaseA (RecA), involved in DNA repair and the SOS response, and PirA, an iron uptake receptor supporting virulence, likely contributes to the reduced pathogenic potential observed in the *G. mellonella* infection model.

Interestingly, the proteomic analysis revealed upregulation of proteins associated with ribosome maturation (RimP), tRNA modification (TrhO), and heme biosynthesis (HemE), indicating a shift toward maintaining essential cellular processes. Enhanced expression of ribosomal proteins (RplC, RplM, RpmF), transcription factors (RpoA), and energy production-related enzymes (AtpD) further supports the prioritization of survival over virulence in the mutant strain. Despite these extensive shifts, no changes were observed in the expression of proteins linked to antibiotic resistance mechanisms, such as efflux pumps or beta-lactamases. The observed variations in antibiotic susceptibility likely result from indirect effects of *smpB* disruption on cellular processes rather than direct alterations in resistance pathways. This finding underscores the specificity of the mutation impact on biofilm, motility, and pathogenicity while preserving the bacterial drug susceptibility profile for most antibiotics.

**Fig 5:**
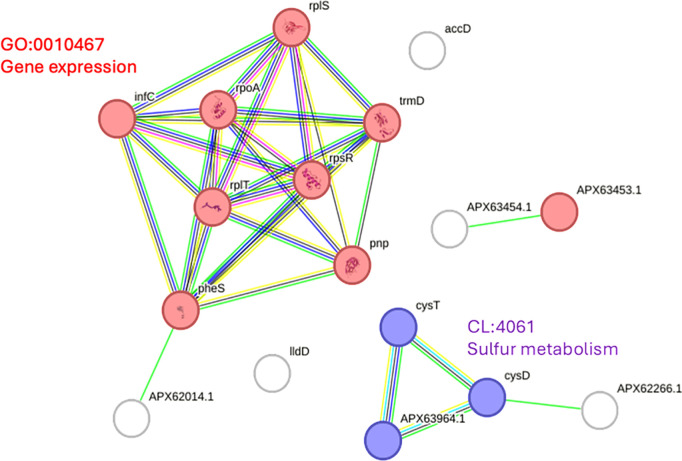
Protein-protein interaction network of upregulated genes in the *Acinetobacter baumannii smpB* mutant. The network was identified using the STRING database. Each node represents a gene or protein, with the size of the node indicating the strength of evidence supporting its interactions. The edges are color-coded to represent different types of interactions: green for gene co-occurrence, blue for co-expression, purple for experimental data, yellow for text-mining evidence, black for protein homology, and light blue for known interactions from curated databases. Key genes in the network include *lntC*, *rpoA*, *rplS*, *trmD*, *rpsR*, *pnp*, *pheS*, *rplT*, *accD*, *cysT*, *cysW*, *lldD*, and several genes identified by specific protein identifiers (APX63453.1, APX63454.1, APX62014.1, APX62266.1, APX63964.1).

**Fig 6:**
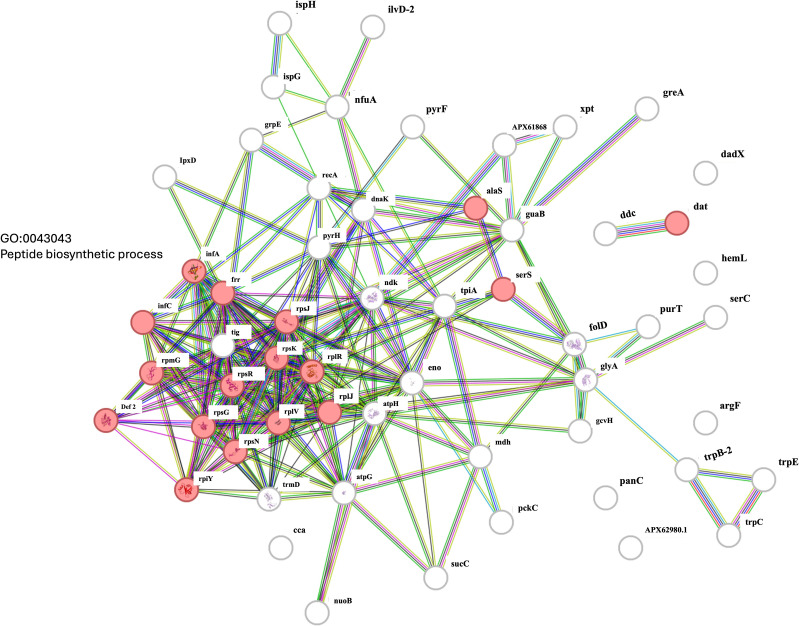
Protein-protein interaction network of downregulated genes involved in the peptide biosynthetic process (GO:0043043) in the *Acinetobacter baumannii smpB* mutant.

Network analysis using the STRING database highlighted the broad impact of *smpB* disruption on essential cellular functions. Downregulated proteins such as RecA, DnaK, and GroEL were embedded in networks associated with stress responses, ribosome function, and protein stability (Fig 5). Upregulated proteins, including RimP, rpoA, and accD, were linked to transcriptional regulation and metabolic homeostasis, reflecting compensatory mechanisms supporting fundamental cellular processes (Fig 6).

These proteomic findings align closely with phenotypic observations, including reduced biofilm formation, attenuated twitching motility, and diminished virulence in the *G. mellonella* infection model. Collectively, the data highlight the critical role of SmpB in coordinating *A. baumannii* pathogenic and adaptive responses, with disruption leading to a marked reduction in its virulence potential.

This network was also identified using the STRING database. As in part, nodes represent genes or proteins, with their sizes corresponding to the strength of evidence supporting their interactions. The edges are similarly color-coded: green for gene co-occurrence, blue for co-expression, purple for experimental data, yellow for text-mining evidence, black fo protein homology, and light blue for known interactions from curated databases. Key genes involved include *infC*, *rplB*, *rpsL*, *rplV*, *tsf*, *serS*, *rlmL*, *recA*, and *dnaK*, which highlight the intricate web of interactions and regulatory pathways associated with these genes in the peptide biosynthetic process.

## 4. Discussion

SmpB, a critical component of the ribosome rescue system, supports protein synthesis under stress conditions. Our study found no significant difference in the growth of *A. baumannii* WT and *smpB* mutant strains under nutrient-rich laboratory conditions, indicating that SmpB is not essential for growth in non-stressful environments. This observation aligns with previous reports suggesting that ribosome rescue mechanisms, including SmpB and tmRNA, are often redundant during exponential growth but become vital under stress or during stationary phase when damaged or truncated mRNAs accumulate [21,22].

The *smpB* mutant exhibited significantly reduced biofilm formation, consistent with earlier studies indicating that defects in trans-translation impair biofilm development [23,24]. Scanning and transmission electron microscopy revealed sparse, disorganized biofilms in the mutant compared to the dense biofilms of the WT, while cell morphology remained unaffected. Proteomic analysis further supported this phenotype, showing downregulation of chaperones such as GroEL and DnaK—key facilitators of protein folding and biofilm structural stability [25–28]. Reduced expression of AtpF and FusA, proteins involved in motility and energy production, likely contributes to impaired surface adherence and matrix development. While swimming and swarming motility were unaffected, the *smpB* mutant displayed significantly reduced twitching motility, consistent with AtpF and FusA downregulation. These findings reinforce the role of SmpB in regulating pili-dependent motility and biofilm-related functions. Motility assays revealed no differences in swimming or swarming, but significantly reduced twitching motility in the *smpB* mutant, consistent with the downregulation of AtpF and FusA. These results support previous findings that SmpB influences post-transcriptional regulation of motility and biofilm-associated traits [29].

Disruption of *smpB* did not significantly alter susceptibility to β-lactam antibiotics such as ampicillin, cefoperazone/sulbactam, imipenem, and meropenem, in line with prior studies emphasizing the role of β-lactamases, efflux pumps, and outer membrane proteins in resistance [30–32]. However, altered responses to piperacillin/tazobactam (increased sensitivity) and cefepime and tetracycline (decreased sensitivity) suggest that SmpB-associated stress responses may indirectly influence antimicrobial susceptibility [33,34]. These changes may reflect altered membrane integrity, possibly involving AccD—a protein involved in fatty acid biosynthesis and membrane stability [35]. Notably, aminoglycoside sensitivity may also be modulated, supporting previous evidence that ribosome-associated mutations can impact susceptibility to this class of antibiotics [36–38]. In the *Galleria mellonella* infection model, the *smpB* mutant showed reduced virulence, although the difference did not reach statistical significance—possibly due to model limitations or sample size. Nonetheless, the observed trend supports the hypothesis that SmpB contributes to pathogenicity under host-like stress conditions [39–41], Reduced biofilm formation, impaired motility, and downregulation of PirA, an iron acquisition receptor essential for in vivo survival, further suggest diminished virulence due to impaired stress adaptation and nutrient uptake.

Compensatory mechanisms appear to be activated in the *smpB* mutant, as shown by the upregulation of RimP and TrhO—proteins involved in ribosome maturation and tRNA modification, respectively [42,43]. In contrast, ribosomal proteins RpsG, RplS, and RpsR were downregulated, indicating possible disruption of ribosomal assembly or stability [44–45]. The downregulation of GroEL and AtpF further supports the observed loss of biofilm integrity and motility [25–28], while increased expression of transcriptional regulators like RpoA may reflect a shift toward maintaining core cellular functions under translational stress [46,47]. STRING network analysis highlighted RecA, DnaK, and GroEL as central proteins linking ribosome function, DNA repair, and protein homeostasis—functions critical for biofilm persistence and environmental resilience. Their downregulation likely compromises the bacterium softility to adapt and survive under stress.

## 5. Conclusion

This study highlights the essential role of SmpB in *A. baumannii* virulence, biofilm formation, motility, and stress response. Its disruption compromises key pathogenic traits without broadly affecting antibiotic resistance. As a component unique to bacteria, SmpB presents a promising therapeutic target for novel antimicrobials, particularly against multidrug-resistant *A. baumannii*. Future studies in mammalian models are needed to validate its clinical potential.

## Supporting information

S1 Fig *smpB* amplicons from mutant *Acinetobacter baumannii.* Agarose gel electrophoresis showing the *smpB* amplicon from the mutant strain (~477 bp). Lane M: 100 bp Plus DNA ladder; Lane 1: *smpB* amplicon.
(TIF)

S2 Fig Sequencing analysis confirming the mutation in the smpB gene of *Acinetobacter baumannii.* The upper sequence shows the wild-type smpB gene, while the lower sequence represents the mutant strain. A point mutation is observed at nucleotide position 212, with cytidine (C) replaced by thymine (T).
(TIF)

S3 Fig Amino acid sequences of the smpB gene in *Acinetobacter baumannii* wild-type and *smpB* mutant strains. The amino acid substitution resulting from the C212T mutation is highlighted: residue 89 changes from arginine (R) to glutamine (Q) in the mutant strain.
(TIF)

S1 Quantitative proteomic dataset comparing wild-type and *smpB* mutant *Acinetobacter baumannii.* This file contains the full list of differentially expressed proteins identified by LC-MS/MS analysis between wild-type and smpB mutant strains. Data include protein IDs, gene names, fold-change values, and associated biological pathways, particularly those related to biofilm formation, motility, and virulence.
(XLSX)

S2 Raw Gel. (TIF)
