# Supplementary figures and images for: CRISPR/Cas9-targeted smpB mutation revealing roles in biofilm formation, motility, and antibiotic susceptibility in Acinetobacter baumannii
Source: PLoS One. 2025 Aug 4;20(8):e0329638. doi: 10.1371/journal.pone.0329638 (PMC12321068; doi:10.1371/journal.pone.0329638)

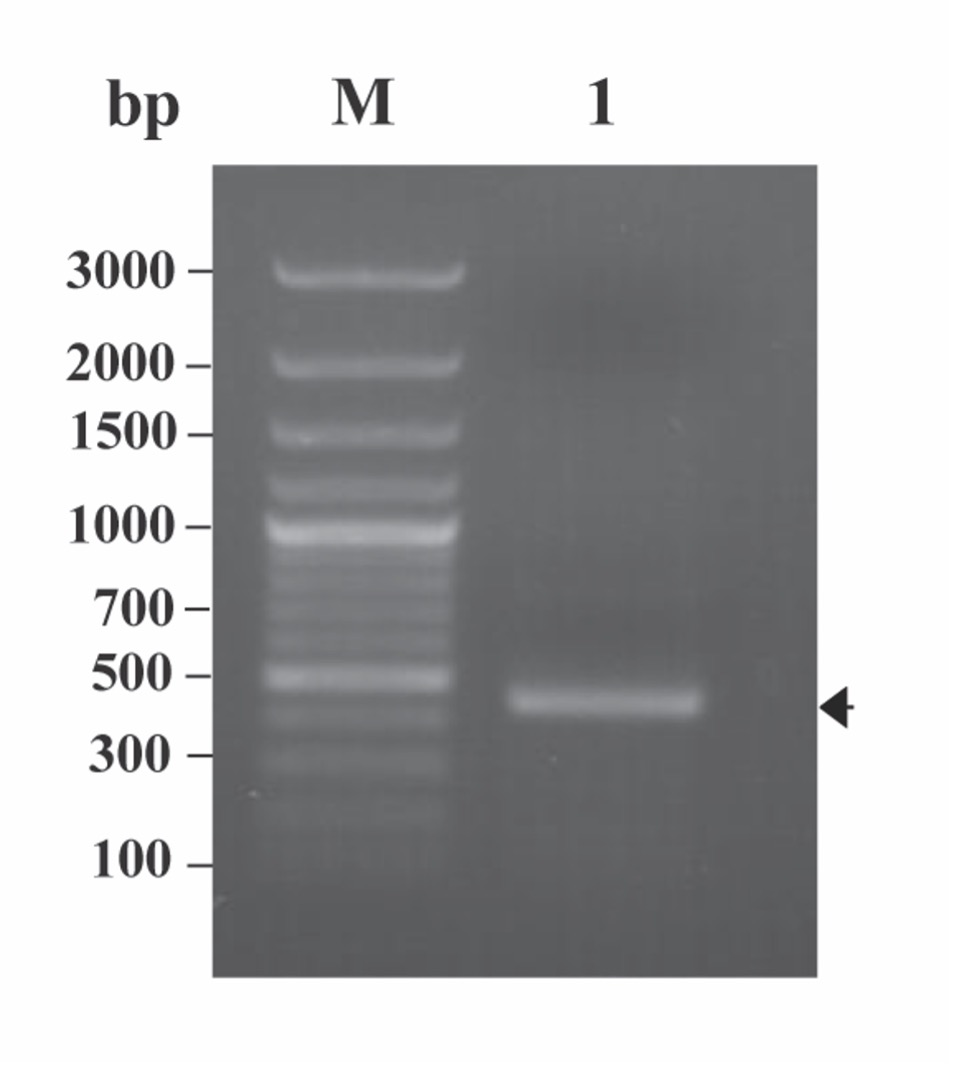

Supplement: S1 Fig — Agarose gel electrophoresis showing the smpB amplicon from the mutant strain (~477 bp). Lane M: 100 bp Plus DNA ladder; Lane 1: smpB amplicon. (TIF) [file pone.0329638.s001.tif]

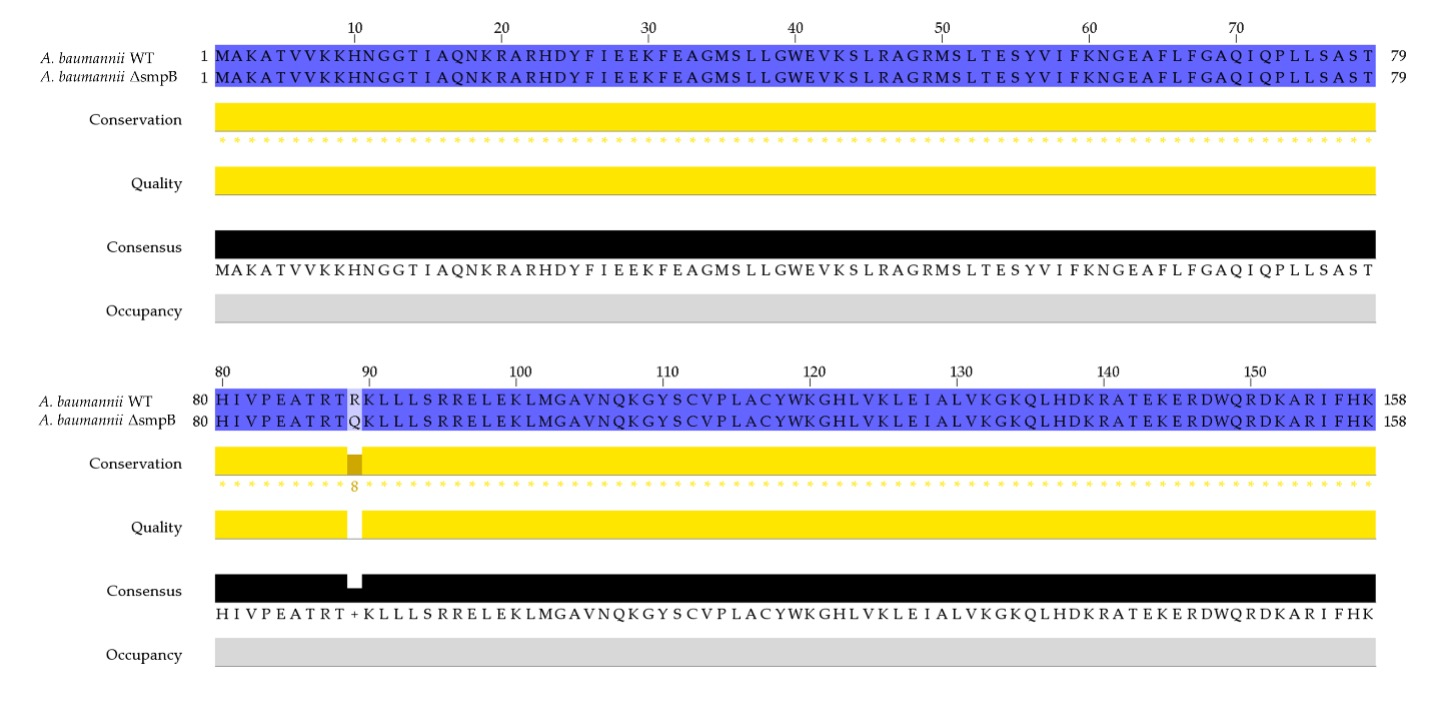

Supplement: S2 Fig — The upper sequence shows the wild-type smpB gene, while the lower sequence represents the mutant strain. A point mutation is observed at nucleotide position 212, with cytidine (C) replaced by thymine (T). (TIF) [file pone.0329638.s002.tif]

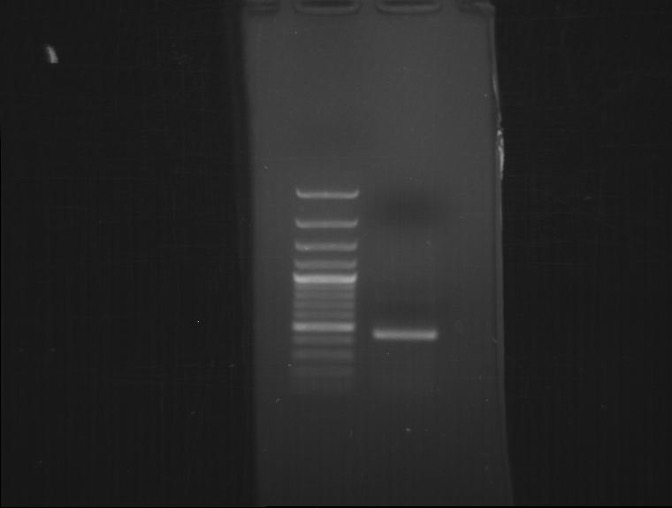

Supplement: S2 — (TIF) [file pone.0329638.s005.tif]
